# Digital religion and hybrid religious authority in algorithmically mediated environments

**DOI:** 10.3389/fsoc.2026.1892383

**Published:** 2026-08-19

**Authors:** Aruzhan Akhmetova, Gulnara Rakhimova, Yershat Onngar, Mukhan Issakhan, Nagima Baitenova

**Affiliations:** 1 Egyptian University of Islamic Culture Nur-Mubarak, Almaty, Kazakhstan; 2 Abai Kazakh National Pedagogical University, Almaty, Kazakhstan; 3 Institute of Experimental and Theoretical Physics, Al-Farabi Kazakh National University, Almaty, Kazakhstan

**Keywords:** constrained pluralization, digital religion, mediatization, platformization, religiosity, religious authority

## Abstract

The rapid expansion of digital communication technologies has transformed the ways religious authority, participation, and legitimacy are produced, circulated, and negotiated within contemporary societies. It aims to develop the concept of constrained pluralization as a context-sensitive theoretical model for understanding digital religion within post-Soviet and regulated sociopolitical environments. The study argues that digitally mediated religiosity develops through the interaction of four interconnected mechanisms: platform affordances, algorithmic visibility, institutional legitimacy, and governance structures. The conceptual analysis suggests that digital platforms expand access to religious discourse and diversify communicative participation while the boundaries of legitimate religious authority remain shaped by institutional recognition and regulatory oversight. It argues that digital religion should not be interpreted through binary oppositions between institutional hierarchy and unrestricted digital pluralism. The article contributes to a more globally sensitive and theoretically differentiated understanding of how digital technologies reorganize religious authority, participation, and legitimacy across varying sociopolitical contexts.

## Introduction

Social media platforms, algorithmically curated communication environments, and networked forms of interaction increasingly structure everyday religious engagement by enabling new modes of participation that go far beyond the traditional institutional settings. In this sense, digital religion has become a leading interdisciplinary field focusing on the blending of religious practice offline and online, on the influence of digital media on religious life, and the conditions of communicating within a network (Campbell H., 2012; Campbell H. A., 2012). Instead of just serving as passive channels of communication, digital platforms influence the visibility, availability, and credibility of religious messages by incorporating the specific communicative styles of the platforms, the underlying algorithmic systems, and the patterns of user interactions.

Existing scholarship has primarily focused on how digital environments change religious communication, authority, and collective identity formation. Studies on digital religion show that networked media environments not only offer the general public increased access to religious knowledge but also enable a wide range of religious actors, such as independent preachers, digital influencers, and content creators, to engage in religious discourse outside of traditional institutional hierarchies. These changes have largely been examined from two main theoretical perspectives. The first sees the democratization of religious authority as digital media challenge institutional monopolies by increasing interpretive participation and communicative accessibility. The second identifies fragmentation and decentralization as outcomes driven by digital communication leading to the weakening of institutional authority and the rise of more individualized and fluid forms of religiosity (Hjarvard, 2011). Complementing these approaches, scholarship on networked religion highlights the emergence of participatory and translocal religious communities structured through digitally mediated interaction and communicative connectivity.

Although these perspectives provide important insights into the transformation of religion under conditions of digital mediation, they remain largely grounded in Western and comparatively liberal sociopolitical contexts in which religious institutions operate under relatively limited regulatory oversight. Consequently, prevailing theoretical frameworks insufficiently explain how digital religion develops in environments where religious life continues to be strongly shaped by institutional authority, governance structures, and state regulation. In such contexts, digital media do not simply pluralize religious authority in unrestricted ways; rather, digitally mediated religious participation unfolds within historically and politically structured boundaries that regulate legitimacy, visibility, and acceptable forms of religious expression (Ibrahim, 2022).

The current theories have significantly advanced the study of digital religion but only partially explain religious dynamics in regulated sociopolitical contexts. Mediatization, networked religion, hybrid religious authority, platformization, and negotiated authority each explain how digital media reshape communication, participation, visibility, or legitimacy. However, they largely assume that digital transformation is driven by media logics, institutional adaptation, or participatory interaction, paying limited attention to how governance structures regulate legitimate religious expression. This leaves an important conceptual gap. In regulated environments, digital platforms diversify religious communication, yet this diversification remains constrained by institutional recognition and state regulation. Existing frameworks do not adequately explain how these governance mechanisms shape the production, circulation, and legitimacy of digital religious authority. The concept of constrained pluralization addresses this gap by integrating insights from existing theories while treating governance as a constitutive rather than contextual dimension of digital religion. It explains how platform affordances, algorithmic visibility, institutional legitimacy, and governance regulation interact to produce context-specific forms of religious authority. Its added explanatory value lies in accounting for why similar digital platforms generate different patterns of religious pluralization across regulated and liberal sociopolitical settings.

Post-Soviet societies, particularly Kazakhstan, provide an analytically valuable context for reconsidering existing theories of digital religion. These settings reveal how expanding digital participation coexists with strong institutional regulation and state oversight, exposing theoretical dynamics that remain insufficiently explained by prevailing models. The growing use of platforms such as Instagram, YouTube, Telegram, and TikTok has transformed them into communication infrastructures for accessing religious knowledge, interpret religious teachings, and participate in religious life. Besides, they also exist along with the old institutional arrangements (Zabirova et al., 2025).

This article argues that existing theories of digital religion insufficiently account for the structured and context-dependent nature of religious diversification in regulated sociopolitical environments. To address this limitation, the article develops the concept of *constrained pluralization* of religious authority. Constrained pluralization refers to a form of digitally mediated religious diversification in which digital platforms expand access to religious actors, interpretations, and modes of communicative participation, while the boundaries of legitimate authority continue to be shaped by institutional recognition and regulatory governance. Unlike approaches that interpret digital religion primarily through the lenses of democratization or fragmentation, constrained pluralization emphasizes the coexistence of diversification and structural continuity within digitally mediated religious environments.

Drawing on scholarship in digital religion, mediatization theory, and networked communication, the article advances a context-sensitive theoretical framework for understanding contemporary religiosity in post-Soviet digital environments. The analysis proposes four interconnected mechanisms through which constrained pluralization emerges: (1) platform affordances that expand communicative participation; (2) algorithmic visibility that redistributes symbolic influence; (3) institutional legitimacy that stabilizes acceptable forms of religious authority; and (4) governance structures that regulate the boundaries of legitimate religious expression. These four mechanisms should not be understood as independent variables but as an interacting system. Platform affordances create opportunities for religious participation by enabling content production, interaction, and dissemination. Algorithmic visibility selectively amplifies some actors and messages over others, thereby shaping patterns of communicative influence. However, algorithmic prominence alone does not confer religious authority. Institutional authority conditions whether digitally visible actors are recognized as credible within established religious traditions, while governance structures define the legal and political boundaries within which both platforms and religious institutions operate. Governance therefore moderates the effects of platform affordances and algorithmic visibility by regulating acceptable forms of religious expression, whereas institutional recognition mediates the translation of digital visibility into recognized religious authority.

The article makes three principal theoretical contributions. First, it introduces constrained pluralization as a conceptual framework for explaining how digital media reorganize religious authority through simultaneous processes of diversification and institutional regulation. Second, it extends existing theories of digital religion by demonstrating the central role of governance structures and institutional credibility in shaping digitally mediated religious participation. Third, it contributes to ongoing efforts to decenter Western-centered approaches in the study of religion and digital media by foregrounding the historically specific dynamics of post-Soviet religious transformation. Through this framework, the article seeks to advance a more globally sensitive understanding of how digital technologies restructure religiosity across diverse political, institutional, and communicative contexts.

### Research methods

This article adopts a conceptual and theory-building research design aimed at developing a context-sensitive framework for understanding digital religiosity within regulated sociopolitical environments. Rather than seeking bounded empirical generalization, the study advances an interpretive and analytical synthesis of interdisciplinary scholarship on digital religion, mediatization, platformization, and networked communication in order to theorize how digital media reorganize religious authority, participation, and legitimacy under conditions of technological mediation.

The analytical framework draws on an integrative synthesis of scholarship in the sociology of religion, media and communication studies, and digital culture. The existing theories have significantly advanced understanding of how digital media reshape religious communication, authority, and community formation, and therefore they have largely been developed in Western liberal contexts where religious pluralization operates under relatively limited institutional and regulatory constraints. As a result, these approaches primarily emphasize decentralization, democratization, and individualization, while giving comparatively less attention to the role of governance structures and institutional authority in shaping digitally mediated religious life.

To address this limitation, the article develops the concept of *constrained pluralization* of religious authority as a context-sensitive analytical framework for understanding religious diversification under conditions of institutional continuity and regulatory governance. Constrained pluralization conceptualizes digital religion as the result of four mutually reinforcing mechanisms rather than as the effect of any single factor. Platform affordances expand opportunities for religious participation, algorithmic visibility redistributes communicative influence determines which actors acquire recognized authority, and governance structures regulate the boundaries of acceptable religious expression. Their relationship is neither strictly sequential nor hierarchical; instead, they operate recursively. Platform affordances enable participation, algorithms shape visibility evaluates credibility, and governance influences all three by establishing the regulatory conditions within which digital religious communication occurs.

In this paper Kazakhstan functions as an illustrative analytical case that demonstrates how the proposed framework operates in practice. The objective is not to test theory empirically but to examine the explanatory value of constrained pluralization through an existing body of scholarship on religious governance, digital media, and Islam in Kazakhstan. The framework is developed through the synthesis of existing scholarship on digital religion, mediatization, platformization, and religious governance, while observations from Kazakhstan and comparable post-Soviet settings provide an illustrative context that highlights limitations in prevailing theories. The purpose is therefore not to derive theory inductively from a single national case, but to develop a context-sensitive framework with broader analytical relevance for understanding digitally mediated religion in regulated sociopolitical environments.

The article consequently advances a theory-building intervention that seeks to extend contemporary understandings of digital religion beyond dominant Western-centered models. By integrating perspectives from digital religion studies, mediatization theory, and post-Soviet religious governance, the study develops more globally sensitive conceptualization of how digital technologies reorganize the production, circulation, negotiation, and regulation of religious authority in contemporary societies.

### Expansion of digital religious practices

The development of digital media has profoundly changed the communication settings through which religious knowledge, participation, and engagement are organized in the modern world. Research on digital religion considers digital platforms not simply as means of transmitting religious information, but as socio-technical infrastructures that influence the distribution, accessibility, and understanding of religious discourse within networked communication environments (Lövheim and Lynch, 2011). Platformization research shows that social media platforms privilege particular forms of communication: immediacy, visibility, accessibility, and continuous interaction (Grigore and Cobzeanu, 2025). In religious settings, the logic of the platform encourages the spread of micro-sermons, theological teachings visually made simple, engaging discussions, religious contents being given to users by the algorithms all these are the characteristics of the fast consumption in digital media that are wrapped up in the content being spread through the platform. The significance of these developments lies not only in the increased availability of religious materials, but also in the reconfiguration of the communicative architecture through which religious engagement becomes socially organized. Religious communication consequently becomes integrated into continuously accessible and highly networked media environments rather than remaining confined to temporally bounded institutional settings.

Mediatization scholarship also suggests that digital communication reshapes the timing and continuity of religious participation (Hjarvard, 2016). In the social media religious content is hidden in the casual habits of digital media consumption next to entertainment, lifestyle, and informational content. It implies a shift from the religious engagement in episodic forms that is traditionally linked to localized institutional encounters and toward the forms of mediated exposure that are ambient and continuously circulating. From a conceptual standpoint, this change simply reflects the rearrangement of the communicative conditions of religiosity rather than the shedding of institutional religion itself.

Studies of digital religion additionally emphasize the diversification of interpretive access enabled by networked communication environments. Digital media facilitate flexible and user-oriented forms of religious learning by expanding access to multiple religious actors, interpretive styles, and communicative perspectives (Campbell and Tsuria, 2021). Digital mediation effectively extends opportunities for people to engage selectively with religious discourse, at the same time it diminishes the exclusiveness of officially established means of delivering religious teaching inside this framework.

The expansion of digital religious practices also raises questions about the relationship between communicative visibility and religious influence. Algorithmically curated platforms privilege content that sustains circulation, interaction, and audience engagement, thereby affecting which religious actors and messages receive greater exposure (Evolvi, 2022). However, visibility should not be equated with recognized religious authority, since its translation into legitimacy depends on institutional and regulatory conditions discussed in the following section.

### Negotiating religious authority in digital environments

Building on the expanded communicative opportunities discussed above, algorithmic visibility reshapes which religious actors gain attention, while institutional recognition determines whether digital prominence is translated into recognized authority. Consequently, religious authority in digitally mediated environments emerges through the interaction of platform visibility, audience engagement, and institutional legitimacy rather than through institutional hierarchy alone (Bunt, 2018). As Selby and Sayeed (2023) argue, algorithmically curated platforms privilege religious actors who sustain audience engagement and communicative responsiveness, making platform-compatible communication an increasingly important source of religious influence.

The transformation of religious authority additionally entails a reconfiguration of audience relations within networked communication systems. Campbell (2020) suggests that digital sphere expose users to a multiplicity of religious actors and interpretive perspectives, thereby expanding opportunities for comparison, selective engagement, and reflexive evaluation. Consequently, authority is increasingly negotiated through communicative interaction rather than accepted solely through institutional position.

Recommendation systems, engagement metrics, and other mechanisms of algorithmic amplification influence which religious actors and interpretations become prominent in digital environments. Platform reach may function as a form of symbolic capital by strengthening perceptions of accessibility, relevance, and authenticity (Andok, 2024). Nevertheless, digital prominence alone does not constitute durable religious authority. Institutional affiliation, theological credentials, and recognition within established traditions remain important markers of credibility.

Digitally mediated religious authority should therefore be understood as hybrid and relational. Algorithmic visibility expands communicative influence, while institutional credibility determines whether such influence is converted into recognized and durable authority. Governance structures further condition this relationship by defining the legal and political boundaries within which religious discourse circulates (Khattak, 2024). Religious authority consequently emerges from the interaction of platform visibility, institutional recognition, and regulatory oversight rather than from any single technological or institutional source.

Changes in religious authority also depend on larger governance mechanisms that determine which forms of religious expression are allowed within certain sociopolitical settings. Research on regulated religions shows that online participation often emerges under the supervision of institutions and state-centered religious governance systems. The dissemination of religious discourse in post-Soviet contexts, for example, takes place through the interaction of increasing digital communication on the one hand, and the long-standing institutional regulation on the other. It challenges the idea that equating digital mediation with radical religious decentralization is the only way to interpret them, as they show the continuing importance of governance mechanisms in the composition of the legitimacy of religious actors, practices, and interpretations. Hoover and Echchaibi (2014) claim that religious authority should therefore be understood as the outcome of the interaction between algorithmic visibility and institutional legitimacy. While digital platforms redistribute communicative influence, institutional recognition continues to determine which actors acquire durable religious authority. This interaction provides the foundation for the broader framework of constrained pluralization developed later in the article.

### Hybridization of religiosity under digital mediation

Research on digital religion increasingly conceptualizes religiosity as emerging across interconnected mediated and institutional environments rather than within separate online and offline domains. Hybrid religiosity refers to the integration of digital interaction, institutional practice, everyday communication, and networked participation into shared communicative structures. Rather than simply describing the coexistence of online and offline religious activity, it captures the reorganization of how religious participation is experienced, negotiated, and socially organized (Zhanabayeva et al., 2026).

Digital communication infrastructures extend religious engagement beyond localized and temporally bounded institutional settings. Sermons, scriptural interpretations, religious discussions, and educational materials now circulate continuously through social media, messaging applications, and video-sharing platforms, embedding religious practices within everyday digital routines. This transformation expands the communicative settings in which institutional and non-institutional forms of religiosity intersect without necessarily displacing established religious institutions.

Hybrid religiosity is further shaped by participatory practices such as content sharing, reposting, peer interpretation, and interactive discussion. These forms of engagement enable religious meaning to be negotiated across dispersed networks where institutional teaching, interpersonal interaction, and mediated communication converge. At the same time, digital mediation fosters new forms of religious belonging. As Bognár (2024) argues, communicative connectivity enables translocal communities to develop through ongoing interaction, symbolic exchange, and shared interpretation across networked environments.

Platformization research further demonstrates that religious discourse has become embedded within ordinary patterns of digital media consumption. Algorithmically curated environments position religious content alongside entertainment, lifestyle, and informational media, creating persistent but often low-intensity forms of religious exposure (Nurfitria, 2023). Nevertheless, institutional organizations continue to retain authority in ritual, doctrinal, and formal religious domains (Pfadenhauer, 2016). Hybrid religiosity should therefore be understood as a negotiated configuration in which digital participation complements rather than replaces institutional authority.

In regulated sociopolitical environments, this hybrid configuration is additionally shaped by governance structures that define the boundaries of legitimate religious participation and expression. The development of digitally mediated religiosity therefore reflects the interaction of platform infrastructures, institutional authority, and regulatory oversight. From this perspective, hybrid religiosity is best understood as a relational phenomenon produced through the continuous interplay of mediated communication, institutional structures, and governance rather than through a rigid distinction between “online” and “offline” religion.

### Platformized religious socialization and youth religiosity

The digitalization of faith among younger generations reflects a broader transformation in the communicative processes through which religious knowledge, legitimacy, and participation are produced. Rather than relying primarily on formal religious instruction or institutional participation, youth religiosity increasingly develops through platformized and networked forms of socialization shaped by media infrastructures, algorithmic systems, and everyday digital interaction (Ma’rof and Abdullah, 2026). Platforms such as TikTok, Instagram, YouTube, and Telegram therefore function not merely as channels of communication but as socio-technical environments in which younger users encounter, interpret, and circulate religious discourse.

Digital media reorganize religious learning by embedding religious content within routine patterns of media consumption alongside entertainment, lifestyle, and informational content. Aslan (2024) characterizes these environments as episodic, non-linear, and personalized, reflecting forms of knowledge acquisition shaped by platform logics and user engagement. Consequently, younger audiences increasingly encounter religious teachings through short-form videos, algorithmically recommended content, and visually simplified theological explanations designed to maximize accessibility and interaction (Fakhruroji, 2025). Religious socialization thus becomes increasingly structured by systems of algorithmic exposure rather than exclusively by institutional transmission.

Although digital platforms expand opportunities for selective engagement with religious content, religious meaning-making remains fundamentally social. Interpretation, legitimacy, and authority are negotiated through peer interaction, online communities, reposting practices, and collaborative discussion rather than transmitted unilaterally by religious institutions (Rozehnal, 2022). This challenges assumptions that digital religion necessarily produces individualized or detached forms of belief. Instead, authority increasingly emerges through communicative visibility, peer validation, and ongoing processes of comparison and interpretive negotiation within networked environments (Aslan and Yildiz, 2024).

These developments are particularly significant in post-Soviet societies, where the revival of public religiosity has coincided with rapid digitalization and generational change (Kaliyeva et al., 2025). Digital platforms have expanded access to religious knowledge while simultaneously raising new questions about legitimacy, credibility, and religious expertise. In Kazakhstan, for example, younger users navigate a digital environment in which algorithmically mediated religious communication intersects with established institutional authority and regulatory oversight (Kerim et al., 2025). Platformized religious socialization should therefore be understood as a process through which religious knowledge and legitimacy are negotiated within digitally mediated communication systems rather than transmitted exclusively through stable institutional hierarchies.

### Constrained pluralization as a theoretical model of digital religion

Constrained pluralization is proposed as a higher-order analytical framework that specifies how established mechanisms of digital religion interact under conditions of institutional and regulatory oversight. Mediatization, networked religion, hybrid authority, platformization, and negotiated authority illuminate important aspects of digital religious communication, participation, and legitimacy (Astor et al., 2024).

The framework addresses this limitation by explaining religious pluralization as the outcome of an interacting system. These mechanisms do not operate independently or in a strictly sequential manner. Their interaction determines whether expanded digital participation produces broader recognition, temporary visibility, or regulatory restriction.

The distinctive contribution of constrained pluralization therefore lies in explaining why comparable digital technologies generate different configurations of religious authority across sociopolitical contexts. In such environments, increased digital participation expands religious discourse while institutional authority continues to shape legitimate recognition.

Studies of digital communication demonstrate that algorithmically structured environments privilege forms of discourse capable of sustaining circulation, responsiveness, and audience engagement (Grigore and Cobzeanu, 2025). Berger and Golan (2024) similarly argue that online religious learning environments reorganize epistemic authority through digitally mediated forms of self-socialization in which users actively negotiate credibility across distributed communicative networks. Nevertheless, visibility alone does not fully determine legitimacy. Institutional affiliation, theological credentials, and recognized religious traditions continue to function as stabilizing mechanisms that regulate acceptable forms of authority and interpretation.

The model additionally contributes to theories of digital religion by foregrounding governance structures as constitutive dimensions of digitally mediated religiosity. However, regulated sociopolitical contexts demonstrate that digital religious participation often unfolds within institutional and legal frameworks that continue to define the boundaries of acceptable religious expression (Ibrahim, 2022). Processes of mediatization within Islamic digital environments remain closely intertwined with institutional and sociopolitical structures that shape the circulation, normalization, and legitimacy of religious discourse (Setianto, 2026).

The concept of constrained pluralization also contributes to broader discussions concerning the relationship between digital mediation and religious authority. The framework developed in this article conceptualizes authority as relationally negotiated across overlapping communicative, and regulatory domains. Atallah (2026), for example, discusses that digitally mediated fatwa authority operates through hybrid configurations in which online circulation and institutional recognition remain deeply interconnected. Religious authority consequently emerges through the dynamic interaction of these interconnected dimensions rather than through singular institutional or technological determinants.

**Figure 1:**
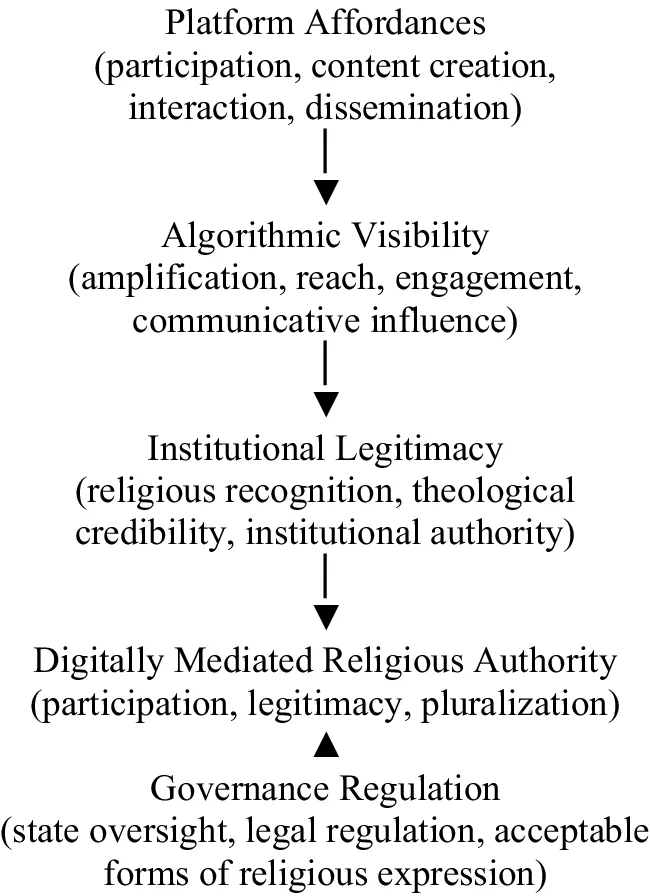
Conceptual framework of constrained pluralization in digitally mediated religion.

Figure 1 summarizes the conceptual framework developed in this article. Although presented sequentially for analytical clarity, the four mechanisms should not be interpreted as a linear causal chain. Instead, constrained pluralization emerges through continuous interaction among platform affordances, algorithmic visibility, institutional legitimacy, and governance regulation.

The process begins with *platform affordances*, which expand opportunities for religious participation by enabling users to create, disseminate, and interact with religious content. These affordances generate a diversified communicative environment in which institutional organizations, independent religious actors, and ordinary users can all participate in digital religious discourse. Algorithmic visibility subsequently determines which actors and messages receive greater public exposure by prioritizing content according to engagement, interaction, and platform-specific recommendation systems. However, increased visibility does not automatically translate into recognized religious authority.

The conversion of digital visibility into religious legitimacy depends upon institutional legitimacy. Religious institutions evaluate theological credibility, recognize qualified religious authorities, and distinguish institutionally accepted interpretations from alternative forms of religious expression. Consequently, institutional legitimacy mediates the relationship between algorithmic visibility and recognized religious authority by determining whether digitally prominent actors acquire durable religious credibility.

*Governance regulation* operates across all three preceding mechanisms rather than functioning as a separate sequential stage. Through legal regulation, institutional oversight, religious policy, and the recognition of official religious organizations, governance establishes the structural conditions within which digital religious communication occurs. It therefore modifies platform affordances by defining the institutional environment in which religious participation takes place, while simultaneously influencing algorithmic visibility by shaping which religious actors, organizations, and forms of religious communication acquire institutional recognition and public legitimacy. Governance thus acts as a downward structural modifier of algorithmic visibility because the legal and institutional framework conditions how digital prominence is translated into socially recognized religious authority.

The relationships among these mechanisms are therefore recursive. Greater platform participation generates new patterns of algorithmic visibility; algorithmic visibility creates opportunities for new religious actors to emerge; institutional legitimacy evaluates the credibility of these actors; and governance continuously shapes the legal and institutional environment within which these interactions occur. The outcome is neither unrestricted digital pluralization nor complete institutional continuity, but a context-specific process of constrained pluralization in which diversification remains structured by governance and institutional authority.

The framework additionally intersects with scholarship emphasizing the embodied and affective dimensions of digitally mediated religiosity. Črnič and Jurekovič (2026) argue that post-pandemic religious practice increasingly unfolds through hybrid configurations combining embodied participation and digital mediation, thereby complicating rigid distinctions between physical and mediated religious experience. Digital religion is embedded within pervasive socio-spatial and affective environments through which religious meaning is continuously negotiated across everyday communicative contexts (Gao et al., 2024). These developments reinforce the argument that digital religion should not be understood as detached from lived social experience, but rather as integrated into broader structures of interaction, embodiment, and mediated participation.

Theoretical discussions of digital theology highlight the necessity of analytically differentiated models that are able to explain the interrelation between technological mediation and institutional continuity. Digital religion offers a new impetus to the development of new theological and communicative structures formed through the incorporation of religious discourse in digitally networked environments. The concept of constrained pluralization extends these discussions by emphasizing that such transformations remain structured through unequal systems of legitimacy, visibility, and governance rather than unfolding solely through unrestricted forms of communicative openness (Zaluchu, 2024).

Constrained pluralization provides a context-sensitive framework for understanding religious diversification within post-Soviet settings. Post-Soviet societies exemplify types of digital religiosity where increasing availability of religious discourse alongside historically embedded systems of institutional oversight and state regulation (Zabirova et al., 2025). This article considers Kazakhstan not merely as an isolated empirical case study, but rather as a meaningful context through which the general theoretical dynamics of regulated digital religiosity can be exposed. This method, among other things, helps to destabilize Eurocentric biases in digital religion scholarship by illustrating that the effects of the digital mediation are, in fact, historically and politically contingent.

The theoretical contribution of constrained pluralization ultimately lies in its capacity to integrate diversification, institutional continuity, technological mediation, and governance regulation within a unified analytical framework. Instead of framing digital religion solely through narratives of institutional decline or unrestricted digital pluralism, the model put forward in the present article sees digitally mediated religiosity as a structurally conditioned process unfolding through the four-way interaction of platform infrastructures, communicative involvement, institutional lawfulness, and regulatory governance. Such a viewpoint helps to give a more worldwide oriented and theoretically more discriminating account of how digital tools reform religious dominion, involvement, and lawful recognition in different sociopolitical milieus.

### Constrained pluralization in the Kazakh context

Kazakhstan provides an analytically valuable context for illustrating the dynamics of constrained pluralization because the expansion of digital religious communication has developed alongside a centralized system of religious governance. Rather than replacing institutional authority, digital platforms have become embedded within a regulatory environment in which state institutions and officially recognized religious organizations continue to shape the production and circulation of legitimate religious discourse. This interaction demonstrates why digital religion in regulated sociopolitical environments cannot be adequately explained through theories emphasizing decentralization or unrestricted religious pluralization alone (Kerim and Kurmanaliyev, 2026).

The first mechanism of constrained pluralization concerns platform affordances. Platforms such as Instagram, Telegram, YouTube, and TikTok have substantially expanded opportunities for religious communication by lowering barriers to content creation, dissemination, and interaction. Official religious institutions, independent preachers, educators, and ordinary believers can all participate in producing and circulating religious knowledge. Sermons, short theological explanations, livestreams, question-and-answer sessions, and educational materials increasingly form part of everyday digital media practices, particularly among younger generations. Platform affordances therefore diversify participation and expand access to religious discourse without necessarily transforming the institutional foundations of religious authority.

However, these affordances operate within a governance structure that conditions how digital participation develops. Kazakhstan’s Law on Religious Activity and Religious Associations establishes a comprehensive legal framework for the registration of religious organizations, regulation of missionary activity, theological expertise, and state oversight of religious affairs. The law assigns the authorized state body responsibility for implementing religious policy, conducting theological examinations, coordinating religious organizations, and regulating the production and dissemination of religious materials (Republic of Kazakhstan, 2011). Consequently, religious communication develops within an institutional environment in which legal regulation defines the boundaries of legitimate religious activity rather than within an unrestricted digital marketplace.

The second mechanism concerns algorithmic visibility. Like other social media environments, Instagram, YouTube, TikTok, and Telegram amplify religious content through engagement-based recommendation systems that privilege interaction, responsiveness, and audience engagement. Algorithmic visibility therefore enables new religious actors to reach large audiences regardless of their formal institutional position. Nevertheless, digital prominence alone does not establish recognized religious authority.

Institutional legitimacy remains a central mechanism through which religious authority is stabilized. In Kazakhstan, the Spiritual Administration of Muslims of Kazakhstan (SAMK) occupies a pivotal position within the country’s Islamic institutional landscape. As the officially recognized national Islamic institution, SAMK coordinates mosque administration, appoints imams, issues religious guidance and fatwas, supervises Islamic education, and increasingly disseminates religious knowledge through its official websites and social media platforms. Recent scholarship further demonstrates that the contemporary concept of *Traditional Islam* has become closely associated with SAMK’s role in promoting Hanafi-Maturidi Islam as the normative framework for religious life while reinforcing values of moderation, social harmony, and loyalty to the secular state (Kozhambekov, 2018).

From the perspective of constrained pluralization, governance regulation functions as a structural modifier of platform affordances rather than merely as an external contextual condition. Platform affordances expand opportunities for participation, while algorithmic systems determine which actors become more visible within digital communication environments. Governance, however, establishes the legal and institutional conditions within which these affordances operate. Through legislation, theological expertise, registration procedures, and cooperation with recognized religious institutions, the state shapes the organizational environment in which religious communication takes place (Republic of Kazakhstan, 2011). The Committee for Religious Affairs likewise coordinates state policy on religion, monitors developments in the religious sphere, conducts analytical work, and cooperates with local authorities in implementing religious policy (Committee for Religious Affairs, n.d.). Governance therefore influences not only religious organizations but also the institutional conditions under which digitally mediated religious authority acquires public legitimacy.

This interaction is particularly visible in Kazakhstan’s contemporary digital religious sphere. Official religious institutions have expanded their presence across websites and social media to disseminate sermons, educational materials, fatwas, and public guidance, while independent religious actors increasingly use Instagram, Telegram, YouTube, and TikTok to communicate with broader audiences. Although these platforms diversify access to religious knowledge, users continue to evaluate religious authority through institutional affiliation, theological qualifications, and the credibility of recognized religious organizations. Digital participation therefore coexists with institutional continuity rather than replacing it.

The Kazakh case therefore serves not merely as a contextual example but as an analytical illustration of constrained pluralization. It demonstrates that platform affordances alone do not determine patterns of religious authority. Rather, algorithmic visibility is mediated by institutional legitimacy and conditioned by governance regulation. Digital technologies diversify participation, but the scope and consequences of this diversification remain structured by legal and institutional arrangements. Kazakhstan therefore illustrates the central proposition of constrained pluralization: digital pluralization unfolds within, rather than independently of, systems of governance and institutional authority.

## Conclusion

The expansion of digital communication technologies has transformed the ways religious authority, participation, and legitimacy are negotiated within contemporary societies. While existing theories of digital religion have provided valuable insights into processes of decentralization, democratization, and networked participation, they offer only a partial explanation of religious change in contexts where institutional authority and governance continue to shape legitimate religious expression. This article has addressed this limitation by proposing *constrained pluralization* as a context-sensitive framework for understanding digitally mediated religion in regulated sociopolitical environments.

The constrained pluralization conceptualizes religious diversification as the outcome of interactions among platform affordances, algorithmic visibility, institutional legitimacy, and governance regulation. The framework demonstrates that digital platforms expand opportunities for participation and access to religious discourse, while institutional recognition and regulatory oversight continue to define the boundaries of legitimate authority. Digitally mediated religion therefore develops through negotiated rather than unrestricted processes of pluralization.

By foregrounding Kazakhstan as an illustrative post-Soviet context, the article also contributes to efforts to decenter Western-oriented approaches in the study of digital religion. The Kazakh case demonstrates how expanding digital participation coexists with institutional oversight and state regulation, highlighting theoretical dynamics that remain insufficiently addressed by prevailing models. Although developed with reference to post-Soviet settings, constrained pluralization offers a broader analytical framework for examining digitally mediated religion across other regulated sociopolitical environments.

As future research it is suggested to evaluate and refine the framework through comparative studies of different governance regimes, religious traditions, and platform ecosystems. Such research would clarify how varying configurations of institutional authority, algorithmic visibility, and regulatory governance shape religious communication across diverse political and cultural settings. More broadly, constrained pluralization contributes to debates on digital religion by demonstrating that technological innovation and religious pluralization remain embedded within historically and institutionally structured systems of legitimacy and governance.

## Data Availability

The original contributions presented in the study are included in the article/supplementary material, further inquiries can be directed to the corresponding author.
